# Supplementary material for: Comparative multiomics analysis of cell physiological state after culture in a basket bioreactor
Source: Sci Rep. 2022 Nov 23;12:20161. doi: 10.1038/s41598-022-24687-4 (PMC9686226; doi:10.1038/s41598-022-24687-4)
Supplement: Supplementary file 1 — Supplementary Information 1. [file 41598_2022_24687_MOESM1_ESM.zip › raw data/Transcriptomic raw data/6.Enrichment/2.KEGG/TE1vsTC1/ALL/TE1vsTC1.all_KEGGenrich.html]

Pathway Enrichment


# The most enriched pathway terms

Statistic method: hypergeometric test

FDR correction method: Benjamini and Hochberg

| Term | Description | Sample number | Background number | P-value | Corrected P-value | Gene\_id | Gene\_name | KEGG\_ID |
| --- | --- | --- | --- | --- | --- | --- | --- | --- |
| csab04630 | JAK-STAT signaling pathway | 12/175 | 111/5900 | 9.71931706433595e-05 | 0.0183636567867612 | 103226390 103235305 103237005 103246489 103236305 103218491 103219268 103237430 103247197 103238870 103224900 103223920 | IL6 IL11 IL7 CCND1 IL15 PTPN6 CNTFR MYC MCL1 SOCS2 PIK3R3 IL6R | csab:103226390 csab:103235305 csab:103237005 csab:103246489 csab:103236305 csab:103218491 csab:103219268 csab:103237430 csab:103247197 csab:103238870 csab:103224900 csab:103223920 |
| csab04060 | Cytokine-cytokine receptor interaction | 15/175 | 170/5900 | 0.000139118612020918 | 0.0183636567867612 | 103226390 103235305 103241278 103246725 103237005 103236305 103219268 103235789 103225587 103242551 103242198 103225819 103223920 103221402 103242710 | IL6 IL11 IL1B BMP2 IL7 IL15 CNTFR CXCL1 TNFRSF1B TNFRSF6B CXCL16 TNFRSF18 IL6R TNFRSF21 CCL2 | csab:103226390 csab:103235305 csab:103241278 csab:103246725 csab:103237005 csab:103236305 csab:103219268 csab:103235798 csab:103225587 csab:103242551 csab:103242198 csab:103225819 csab:103223920 csab:103221402 csab:103242710 |
| csab04621 | NOD-like receptor signaling pathway | 12/175 | 131/5900 | 0.000466895874830619 | 0.0393892729204762 | 103226390 103224057 103241278 103224476 103239154 103230198 103235789 103224480 103240296 103215179 103242710 103239156 | IL6 LOC103224057 IL1B LOC103224476 OAS1 IKBKE CXCL1 LOC103224480 TNFAIP3 CARD6 CCL2 OAS2 | csab:103226390 csab:103224057 csab:103241278 csab:103224476 csab:103239154 csab:103230198 csab:103235798 csab:103224480 csab:103240296 csab:103215179 csab:103242710 csab:103239156 |
| csab04668 | TNF signaling pathway | 10/175 | 98/5900 | 0.000596807165461761 | 0.0393892729204762 | 103226390 103241278 103222156 103236305 103235789 103225587 103218917 103240296 103224900 103242710 | IL6 IL1B EDN1 IL15 CXCL1 TNFRSF1B TRAF1 TNFAIP3 PIK3R3 CCL2 | csab:103226390 csab:103241278 csab:103222156 csab:103236305 csab:103235798 csab:103225587 csab:103218917 csab:103240296 csab:103224900 csab:103242710 |
| csab05162 | Measles | 10/175 | 110/5900 | 0.00147123548534845 | 0.0776812336263979 | 103226390 103241278 103246489 103239154 103230198 103240296 103224900 103223677 103228406 103239156 | IL6 IL1B CCND1 OAS1 IKBKE TNFAIP3 PIK3R3 HSPA6 CBLB OAS2 | csab:103226390 csab:103241278 csab:103246489 csab:103239154 csab:103230198 csab:103240296 csab:103224900 csab:103223677 csab:103228406 csab:103239156 |
| csab05171 | Coronavirus disease - COVID-19 | 12/175 | 163/5900 | 0.00313485250328269 | 0.137933510144438 | 103226390 103241278 103221732 103244654 103221259 103239154 103230198 103224900 103233571 103223920 103242710 103239156 | IL6 IL1B LOC103221732 HBEGF RPL22L1 OAS1 IKBKE PIK3R3 CFD IL6R CCL2 OAS2 | csab:103226390 csab:103241278 csab:103221732 csab:103244654 csab:103221259 csab:103239154 csab:103230198 csab:103224900 csab:103233571 csab:103223920 csab:103242710 csab:103239156 |
| csab04657 | IL-17 signaling pathway | 7/175 | 69/5900 | 0.00410370260609584 | 0.154768212572757 | 103226390 103241278 103230198 103235789 103240296 103242634 103242710 | IL6 IL1B IKBKE CXCL1 TNFAIP3 TRAF4 CCL2 | csab:103226390 csab:103241278 csab:103230198 csab:103235798 csab:103240296 csab:103242634 csab:103242710 |
| csab05418 | Fluid shear stress and atherosclerosis | 9/175 | 111/5900 | 0.00544641070691229 | 0.174086453773731 | 103241278 103243277 103215685 103222156 103220724 103218213 103218702 103224900 103242710 | IL1B ITGB3 PLAT EDN1 SDC1 GPC1 MGST1 PIK3R3 CCL2 | csab:103241278 csab:103243277 csab:103215685 csab:103222156 csab:103220724 csab:103218213 csab:103218702 csab:103224900 csab:103242710 |
| csab04640 | Hematopoietic cell lineage | 6/175 | 56/5900 | 0.005934765469559 | 0.174086453773731 | 103226390 103235305 103241278 103243277 103237005 103223920 | IL6 IL11 IL1B ITGB3 IL7 IL6R | csab:103226390 csab:103235305 csab:103241278 csab:103243277 csab:103237005 csab:103223920 |
| csab00590 | Arachidonic acid metabolism | 5/175 | 43/5900 | 0.00837929475293203 | 0.203101103745753 | 103235528 103237812 103245108 103218549 103220573 | PLA2G4C LOC103237812 LTC4S CBR3 PLB1 | csab:103235528 csab:103237812 csab:103245108 csab:103218549 csab:103220573 |
| csab05323 | Rheumatoid arthritis | 6/175 | 61/5900 | 0.00898862705077812 | 0.203101103745753 | 103226390 103235305 103241278 103236305 103235789 103242710 | IL6 IL11 IL1B IL15 CXCL1 CCL2 | csab:103226390 csab:103235305 csab:103241278 csab:103236305 csab:103235798 csab:103242710 |
| csab00600 | Sphingolipid metabolism | 5/175 | 44/5900 | 0.00923186835207966 | 0.203101103745753 | 103236153 103224742 103233234 103230017 103219011 | UGT8 PLPP3 SMPD3 DEGS1 UGCG | csab:103236153 csab:103224742 csab:103233234 csab:103230017 csab:103219011 |
| csab04625 | C-type lectin receptor signaling pathway | 7/175 | 83/5900 | 0.0111793291065665 | 0.225198478879245 | 103226390 103241278 103215465 103230198 103216128 103224900 103228406 | IL6 IL1B EGR3 IKBKE EGR2 PIK3R3 CBLB | csab:103226390 csab:103241278 csab:103215465 csab:103230198 csab:103216128 csab:103224900 csab:103228406 |
| csab05222 | Small cell lung cancer | 7/175 | 87/5900 | 0.0142605817455345 | 0.225198478879245 | 103224652 103246489 103237430 103214795 103218917 103242634 103224900 | GADD45A CCND1 MYC COL4A1 TRAF1 TRAF4 PIK3R3 | csab:103224652 csab:103246489 csab:103237430 csab:103214795 csab:103218917 csab:103242634 csab:103224900 |
| csab00900 | Terpenoid backbone biosynthesis | 3/175 | 18/5900 | 0.0150771774205399 | 0.225198478879245 | 103240824 103215216 103223243 | ACAT2 HMGCS1 HMGCR | csab:103240824 csab:103215216 csab:103223243 |
| csab04061 | Viral protein interaction with cytokine and cytokine receptor | 5/175 | 50/5900 | 0.0156266704416213 | 0.225198478879245 | 103226390 103235789 103225587 103223920 103242710 | IL6 CXCL1 TNFRSF1B IL6R CCL2 | csab:103226390 csab:103235798 csab:103225587 csab:103223920 csab:103242710 |
| csab00100 | Steroid biosynthesis | 3/175 | 19/5900 | 0.0175207963428723 | 0.225198478879245 | 103237411 103226567 103215320 | SQLE CYP51A1 FDFT1 | csab:103237411 csab:103226567 csab:103215320 |
| csab05169 | Epstein-Barr virus infection | 10/175 | 157/5900 | 0.0178210183543997 | 0.225198478879245 | 103226390 103224652 103246489 103239154 103230198 103237430 103240296 103224900 103221664 103239156 | IL6 GADD45A CCND1 OAS1 IKBKE MYC TNFAIP3 PIK3R3 LOC103221664 OAS2 | csab:103226390 csab:103224652 csab:103246489 csab:103239154 csab:103230198 csab:103237430 csab:103240296 csab:103224900 csab:103221664 csab:103239156 |
| csab04151 | PI3K-Akt signaling pathway | 15/175 | 280/5900 | 0.0185843588641319 | 0.225198478879245 | 103226390 103238335 103243277 103237005 103235805 103246489 103245773 103235804 103225530 103246479 103237430 103247197 103214795 103224900 103223920 | IL6 NR4A1 ITGB3 IL7 AREG CCND1 SGK2 EREG EPHA2 FGFR3 MYC MCL1 COL4A1 PIK3R3 IL6R | csab:103226390 csab:103238335 csab:103243277 csab:103237005 csab:103235805 csab:103246489 csab:103245773 csab:103235804 csab:103225530 csab:103246479 csab:103237430 csab:103247197 csab:103214795 csab:103224900 csab:103223920 |
| csab05144 | Malaria | 4/175 | 35/5900 | 0.0191197852061143 | 0.225198478879245 | 103226390 103241278 103220724 103242710 | IL6 IL1B SDC1 CCL2 | csab:103226390 csab:103241278 csab:103220724 csab:103242710 |
| csab05417 | Lipid and atherosclerosis | 10/175 | 160/5900 | 0.0200779369210929 | 0.225198478879245 | 103226390 103241278 103240818 103230198 103235789 103234364 103223257 103224900 103223677 103242710 | IL6 IL1B SOD2 IKBKE CXCL1 LDLR NCF4 PIK3R3 HSPA6 CCL2 | csab:103226390 csab:103241278 csab:103240818 csab:103230198 csab:103235798 csab:103234364 csab:103223257 csab:103224900 csab:103223677 csab:103242710 |
| csab00770 | Pantothenate and CoA biosynthesis | 3/175 | 20/5900 | 0.0201720240064781 | 0.225198478879245 | 103218755 103240354 103240351 | BCAT1 LOC103240354 VNN2 | csab:103218755 csab:103240354 csab:103240351 |
| csab00565 | Ether lipid metabolism | 4/175 | 36/5900 | 0.0210251866414717 | 0.225198478879245 | 103236153 103235528 103224742 103220573 | UGT8 PLA2G4C PLPP3 PLB1 | csab:103236153 csab:103235528 csab:103224742 csab:103220573 |
| csab05164 | Influenza A | 8/175 | 116/5900 | 0.0213967890363079 | 0.225198478879245 | 103226390 103241278 103239154 103230198 103224900 103232396 103242710 103239156 | IL6 IL1B OAS1 IKBKE PIK3R3 NXF3 CCL2 OAS2 | csab:103226390 csab:103241278 csab:103239154 csab:103230198 csab:103224900 csab:103232396 csab:103242710 csab:103239156 |
| csab04933 | AGE-RAGE signaling pathway in diabetic complications | 7/175 | 95/5900 | 0.0221667185353399 | 0.225198478879245 | 103226390 103241278 103222156 103246489 103214795 103224900 103242710 | IL6 IL1B EDN1 CCND1 COL4A1 PIK3R3 CCL2 | csab:103226390 csab:103241278 csab:103222156 csab:103246489 csab:103214795 csab:103224900 csab:103242710 |
| csab04610 | Complement and coagulation cascades | 5/175 | 55/5900 | 0.0228053073078815 | 0.225198478879245 | 103229609 103231401 103215685 103221732 103233571 | SERPINA1 ITGAX PLAT LOC103221732 CFD | csab:103229609 csab:103231401 csab:103215685 csab:103221732 csab:103233571 |
| csab00650 | Butanoate metabolism | 3/175 | 21/5900 | 0.0230316626126501 | 0.225198478879245 | 103240824 103215216 103227954 | ACAT2 HMGCS1 ABAT | csab:103240824 csab:103215216 csab:103227954 |
| csab05219 | Bladder cancer | 4/175 | 38/5900 | 0.0251752059093069 | 0.231861234294795 | 103244654 103246489 103246479 103237430 | HBEGF CCND1 FGFR3 MYC | csab:103244654 csab:103246489 csab:103246479 csab:103237430 |
| csab01230 | Biosynthesis of amino acids | 5/175 | 57/5900 | 0.0261886708974728 | 0.231861234294795 | 103242625 103218755 103224624 103218486 103224094 | ALDOC BCAT1 CTH ENO2 PHGDH | csab:103242625 csab:103218755 csab:103224624 csab:103218486 csab:103224094 |
| csab05200 | Pathways in cancer | 20/175 | 428/5900 | 0.0275820080839996 | 0.231861234294795 | 103226390 103220342 103246725 103237005 103224652 103230197 103222156 103246489 103223483 103236305 103246479 103237430 103224207 103214795 103218917 103218702 103242634 103224900 103239621 103223920 | IL6 EPAS1 BMP2 IL7 GADD45A RASSF5 EDN1 CCND1 WNT7B IL15 FGFR3 MYC WNT2B COL4A1 TRAF1 MGST1 TRAF4 PIK3R3 NOTCH1 IL6R | csab:103226390 csab:103220342 csab:103246725 csab:103237005 csab:103224652 csab:103230197 csab:103222156 csab:103246489 csab:103223483 csab:103236305 csab:103246479 csab:103237430 csab:103224207 csab:103214795 csab:103218917 csab:103218702 csab:103242634 csab:103224900 csab:103239621 csab:103223920 |
| csab05206 | MicroRNAs in cancer | 9/175 | 145/5900 | 0.0278435502348667 | 0.231861234294795 | 103214617 103243277 103246489 103246479 103237430 103247197 103224900 103239621 103214788 | SPRY2 ITGB3 CCND1 FGFR3 MYC MCL1 PIK3R3 NOTCH1 IRS2 | csab:103214617 csab:103243277 csab:103246489 csab:103246479 csab:103237430 csab:103247197 csab:103224900 csab:103239621 csab:103214788 |
| csab05205 | Proteoglycans in cancer | 10/175 | 169/5900 | 0.0281043920357327 | 0.231861234294795 | 103243277 103244654 103246489 103223483 103218491 103237430 103220724 103224207 103218213 103224900 | ITGB3 HBEGF CCND1 WNT7B PTPN6 MYC SDC1 WNT2B GPC1 PIK3R3 | csab:103243277 csab:103244654 csab:103246489 csab:103223483 csab:103218491 csab:103237430 csab:103220724 csab:103224207 csab:103218213 csab:103224900 |
| csab04012 | ErbB signaling pathway | 6/175 | 79/5900 | 0.0291478009470351 | 0.233182407576281 | 103235805 103244654 103235804 103237430 103224900 103228406 | AREG HBEGF EREG MYC PIK3R3 CBLB | csab:103235805 csab:103244654 csab:103235804 csab:103237430 csab:103224900 csab:103228406 |
| csab04010 | MAPK signaling pathway | 13/175 | 247/5900 | 0.0312408653350426 | 0.237884610246048 | 103238335 103241278 103216504 103235805 103224652 103235804 103225530 103246479 103237430 103217090 103235528 103225290 103223677 | NR4A1 IL1B DUSP5 AREG GADD45A EREG EPHA2 FGFR3 MYC CACNB4 PLA2G4C MAP3K6 HSPA6 | csab:103238335 csab:103241278 csab:103216504 csab:103235805 csab:103224652 csab:103235804 csab:103225530 csab:103246479 csab:103237430 csab:103217090 csab:103235528 csab:103225290 csab:103223677 |
| csab05160 | Hepatitis C | 8/175 | 125/5900 | 0.0317118989141827 | 0.237884610246048 | 103246489 103239154 103230198 103237430 103234364 103241901 103224900 103239156 | CCND1 OAS1 IKBKE MYC LDLR CLDN1 PIK3R3 OAS2 | csab:103246489 csab:103239154 csab:103230198 csab:103237430 csab:103234364 csab:103241901 csab:103224900 csab:103239156 |
| csab05210 | Colorectal cancer | 6/175 | 81/5900 | 0.0324388104880975 | 0.237884610246048 | 103235805 103224652 103246489 103235804 103237430 103224900 | AREG GADD45A CCND1 EREG MYC PIK3R3 | csab:103235805 csab:103224652 csab:103246489 csab:103235804 csab:103237430 csab:103224900 |
| csab04919 | Thyroid hormone signaling pathway | 7/175 | 105/5900 | 0.0357750155482903 | 0.249848849838139 | 103243277 103232196 103246489 103237335 103237430 103224900 103239621 | ITGB3 SLC16A2 CCND1 MED30 MYC PIK3R3 NOTCH1 | csab:103243277 csab:103232196 csab:103246489 csab:103237335 csab:103237430 csab:103224900 csab:103239621 |
| csab04066 | HIF-1 signaling pathway | 6/175 | 83/5900 | 0.0359630920221563 | 0.249848849838139 | 103226390 103242625 103222156 103218486 103224900 103223920 | IL6 ALDOC EDN1 ENO2 PIK3R3 IL6R | csab:103226390 csab:103242625 csab:103222156 csab:103218486 csab:103224900 csab:103223920 |
| csab00970 | Aminoacyl-tRNA biosynthesis | 4/175 | 43/5900 | 0.0375811869763687 | 0.254395727224649 | 103226274 103219751 103229694 103238568 | GARS1 IARS1 WARS1 MARS1 | csab:103226274 csab:103219751 csab:103229694 csab:103238568 |
| csab05134 | Legionellosis | 4/175 | 44/5900 | 0.0404161188387975 | 0.266746384336064 | 103226390 103241278 103235789 103223677 | IL6 IL1B CXCL1 HSPA6 | csab:103226390 csab:103241278 csab:103235798 csab:103223677 |
| csab00280 | Valine, leucine and isoleucine degradation | 4/175 | 46/5900 | 0.0464417160940115 | 0.299039342654123 | 103218755 103240824 103215216 103227954 | BCAT1 ACAT2 HMGCS1 ABAT | csab:103218755 csab:103240824 csab:103215216 csab:103227954 |
| csab04310 | Wnt signaling pathway | 8/175 | 141/5900 | 0.0575897652361489 | 0.357756650755817 | 103232583 103246489 103223483 103237430 103215672 103231576 103224207 103237471 | GPC4 CCND1 WNT7B MYC SFRP1 TBL1X WNT2B CCN4 | csab:103232583 csab:103246489 csab:103223483 csab:103237430 csab:103215672 csab:103231576 csab:103224207 csab:103237471 |
| csab04068 | FoxO signaling pathway | 7/175 | 117/5900 | 0.0582709696306824 | 0.357756650755817 | 103226390 103240818 103224652 103246489 103245773 103224900 103214788 | IL6 SOD2 GADD45A CCND1 SGK2 PIK3R3 IRS2 | csab:103226390 csab:103240818 csab:103224652 csab:103246489 csab:103245773 csab:103224900 csab:103214788 |
| csab04672 | Intestinal immune network for IgA production | 3/175 | 31/5900 | 0.0628502232355222 | 0.369241303457306 | 103226390 103230184 103236305 | IL6 PIGR IL15 | csab:103226390 csab:103230184 csab:103236305 |
| csab05410 | Hypertrophic cardiomyopathy | 5/175 | 74/5900 | 0.0677883090010279 | 0.369241303457306 | 103226390 103243277 103222156 103217090 103219220 | IL6 ITGB3 EDN1 CACNB4 TPM2 | csab:103226390 csab:103243277 csab:103222156 csab:103217090 csab:103219220 |
| csab05216 | Thyroid cancer | 3/175 | 32/5900 | 0.0678972596683063 | 0.369241303457306 | 103224652 103246489 103237430 | GADD45A CCND1 MYC | csab:103224652 csab:103246489 csab:103237430 |
| csab05146 | Amoebiasis | 5/175 | 75/5900 | 0.0709700177561204 | 0.369241303457306 | 103226390 103241278 103235789 103214795 103224900 | IL6 IL1B CXCL1 COL4A1 PIK3R3 | csab:103226390 csab:103241278 csab:103235798 csab:103214795 csab:103224900 |
| csab05163 | Human cytomegalovirus infection | 9/175 | 173/5900 | 0.0709880984415743 | 0.369241303457306 | 103226390 103241278 103243277 103246489 103237430 103224900 103221664 103223920 103242710 | IL6 IL1B ITGB3 CCND1 MYC PIK3R3 LOC103221664 IL6R CCL2 | csab:103226390 csab:103241278 csab:103243277 csab:103246489 csab:103237430 csab:103224900 csab:103221664 csab:103223920 csab:103242710 |
| csab04213 | Longevity regulating pathway - multiple species | 4/175 | 53/5900 | 0.0712284345496919 | 0.369241303457306 | 103240818 103224900 103214788 103223677 | SOD2 PIK3R3 IRS2 HSPA6 | csab:103240818 csab:103224900 csab:103214788 csab:103223677 |
| csab00450 | Selenocompound metabolism | 2/175 | 15/5900 | 0.0713307063497069 | 0.369241303457306 | 103224624 103238568 | CTH MARS1 | csab:103224624 csab:103238568 |
| csab00592 | alpha-Linolenic acid metabolism | 2/175 | 15/5900 | 0.0713307063497069 | 0.369241303457306 | 103235528 103220573 | PLA2G4C PLB1 | csab:103235528 csab:103220573 |
| csab05224 | Breast cancer | 7/175 | 125/5900 | 0.0772558141456565 | 0.377034943615796 | 103224652 103246489 103223483 103237430 103224207 103224900 103239621 | GADD45A CCND1 WNT7B MYC WNT2B PIK3R3 NOTCH1 | csab:103224652 csab:103246489 csab:103223483 csab:103237430 csab:103224207 csab:103224900 csab:103239621 |
| csab04666 | Fc gamma R-mediated phagocytosis | 5/175 | 77/5900 | 0.0775764991104528 | 0.377034943615796 | 103240591 103235528 103224742 103224900 103224044 | WASF1 PLA2G4C PLPP3 PIK3R3 PIP5K1A | csab:103240591 csab:103235528 csab:103224742 csab:103224900 csab:103224044 |
| csab04936 | Alcoholic liver disease | 6/175 | 101/5900 | 0.0787088098736464 | 0.377034943615796 | 103226390 103241278 103221732 103246489 103230198 103235789 | IL6 IL1B LOC103221732 CCND1 IKBKE CXCL1 | csab:103226390 csab:103241278 csab:103221732 csab:103246489 csab:103230198 csab:103235798 |
| csab05213 | Endometrial cancer | 4/175 | 55/5900 | 0.0793402901338159 | 0.377034943615796 | 103224652 103246489 103237430 103224900 | GADD45A CCND1 MYC PIK3R3 | csab:103224652 csab:103246489 csab:103237430 csab:103224900 |
| csab00591 | Linoleic acid metabolism | 2/175 | 16/5900 | 0.0799771092518356 | 0.377034943615796 | 103235528 103220573 | PLA2G4C PLB1 | csab:103235528 csab:103220573 |
| csab05217 | Basal cell carcinoma | 4/175 | 56/5900 | 0.0835619061442699 | 0.378119177447712 | 103246725 103224652 103223483 103224207 | BMP2 GADD45A WNT7B WNT2B | csab:103246725 csab:103224652 csab:103223483 csab:103224207 |
| csab00830 | Retinol metabolism | 3/175 | 35/5900 | 0.0840917585687396 | 0.378119177447712 | 103236970 103225584 103231285 | RDH10 DHRS3 ALDH1A3 | csab:103236970 csab:103225584 csab:103231285 |
| csab04064 | NF-kappa B signaling pathway | 5/175 | 79/5900 | 0.0845039070811175 | 0.378119177447712 | 103241278 103224652 103235789 103218917 103240296 | IL1B GADD45A CXCL1 TRAF1 TNFAIP3 | csab:103241278 csab:103224652 csab:103235798 csab:103218917 csab:103240296 |
| csab04913 | Ovarian steroidogenesis | 3/175 | 36/5900 | 0.0898250832894003 | 0.395230366473361 | 103234364 103235528 103237812 | LDLR PLA2G4C LOC103237812 | csab:103234364 csab:103235528 csab:103237812 |
| csab04210 | Apoptosis | 6/175 | 107/5900 | 0.0974392242348174 | 0.417972552998034 | 103224652 103247197 103218917 103245241 103224900 103235922 | GADD45A MCL1 TRAF1 CTSH PIK3R3 PTPN13 | csab:103224652 csab:103247197 csab:103218917 csab:103245241 csab:103224900 csab:103235922 |
| csab05332 | Graft-versus-host disease | 2/175 | 18/5900 | 0.0981602207798413 | 0.417972552998034 | 103226390 103241278 | IL6 IL1B | csab:103226390 csab:103241278 |
| csab05230 | Central carbon metabolism in cancer | 4/175 | 60/5900 | 0.101515853461178 | 0.422713827919369 | 103246479 103237430 103224900 103233435 | FGFR3 MYC PIK3R3 SLC7A5 | csab:103246479 csab:103237430 csab:103224900 csab:103233435 |
| csab04218 | Cellular senescence | 7/175 | 134/5900 | 0.102476079495605 | 0.422713827919369 | 103226390 103224652 103230197 103246489 103237430 103237062 103224900 | IL6 GADD45A RASSF5 CCND1 MYC E2F5 PIK3R3 | csab:103226390 csab:103224652 csab:103230197 csab:103246489 csab:103237430 csab:103237062 csab:103224900 |
| csab04115 | p53 signaling pathway | 4/175 | 61/5900 | 0.10626229497972 | 0.425391601269914 | 103225266 103224652 103246489 103236967 | SESN2 GADD45A CCND1 CD82 | csab:103225266 csab:103224652 csab:103246489 csab:103236967 |
| csab05231 | Choline metabolism in cancer | 5/175 | 85/5900 | 0.107160265389538 | 0.425391601269914 | 103240591 103235528 103224742 103224900 103224044 | WASF1 PLA2G4C PLPP3 PIK3R3 PIP5K1A | csab:103240591 csab:103235528 csab:103224742 csab:103224900 csab:103224044 |
| csab04623 | Cytosolic DNA-sensing pathway | 3/175 | 39/5900 | 0.107959232140471 | 0.425391601269914 | 103226390 103241278 103230198 | IL6 IL1B IKBKE | csab:103226390 csab:103241278 csab:103230198 |
| csab05223 | Non-small cell lung cancer | 4/175 | 63/5900 | 0.116051043413086 | 0.450551109721393 | 103224652 103230197 103246489 103224900 | GADD45A RASSF5 CCND1 PIK3R3 | csab:103224652 csab:103230197 csab:103246489 csab:103224900 |
| csab04930 | Type II diabetes mellitus | 3/175 | 41/5900 | 0.120773752220499 | 0.46209087806104 | 103238870 103224900 103214788 | SOCS2 PIK3R3 IRS2 | csab:103238870 csab:103224900 csab:103214788 |
| csab04940 | Type I diabetes mellitus | 2/175 | 21/5900 | 0.127296678747085 | 0.462231085218733 | 103241278 103217907 | IL1B PTPRN | csab:103241278 csab:103217907 |
| csab05143 | African trypanosomiasis | 2/175 | 21/5900 | 0.127296678747085 | 0.462231085218733 | 103226390 103241278 | IL6 IL1B | csab:103226390 csab:103241278 |
| csab00270 | Cysteine and methionine metabolism | 3/175 | 42/5900 | 0.12738213840009 | 0.462231085218733 | 103218755 103224624 103224094 | BCAT1 CTH PHGDH | csab:103218755 csab:103224624 csab:103224094 |
| csab04390 | Hippo signaling pathway | 7/175 | 142/5900 | 0.128232940738869 | 0.462231085218733 | 103246725 103248148 103235805 103246489 103223483 103237430 103224207 | BMP2 DLG2 AREG CCND1 WNT7B MYC WNT2B | csab:103246725 csab:103248148 csab:103235805 csab:103246489 csab:103223483 csab:103237430 csab:103224207 |
| csab04550 | Signaling pathways regulating pluripotency of stem cells | 6/175 | 116/5900 | 0.129564773887069 | 0.462231085218733 | 103219053 103223483 103246479 103237430 103224207 103224900 | KLF4 WNT7B FGFR3 MYC WNT2B PIK3R3 | csab:103219053 csab:103223483 csab:103246479 csab:103237430 csab:103224207 csab:103224900 |
| csab05207 | Chemical carcinogenesis - receptor activation | 7/175 | 146/5900 | 0.14223327756204 | 0.49335356157322 | 103219053 103246489 103214580 103230198 103237430 103218702 103224900 | KLF4 CCND1 KLF5 IKBKE MYC MGST1 PIK3R3 | csab:103219053 csab:103246489 csab:103214580 csab:103230198 csab:103237430 csab:103218702 csab:103224900 |
| csab05225 | Hepatocellular carcinoma | 7/175 | 146/5900 | 0.14223327756204 | 0.49335356157322 | 103224652 103246489 103223483 103237430 103224207 103218702 103224900 | GADD45A CCND1 WNT7B MYC WNT2B MGST1 PIK3R3 | csab:103224652 csab:103246489 csab:103223483 csab:103237430 csab:103224207 csab:103218702 csab:103224900 |
| csab01521 | EGFR tyrosine kinase inhibitor resistance | 4/175 | 69/5900 | 0.147626937283276 | 0.49335356157322 | 103226390 103246479 103224900 103223920 | IL6 FGFR3 PIK3R3 IL6R | csab:103226390 csab:103246479 csab:103224900 csab:103223920 |
| csab04975 | Fat digestion and absorption | 2/175 | 23/5900 | 0.147705911898899 | 0.49335356157322 | 103224742 103240824 | PLPP3 ACAT2 | csab:103224742 csab:103240824 |
| csab00980 | Metabolism of xenobiotics by cytochrome P450 | 3/175 | 45/5900 | 0.147945082996778 | 0.49335356157322 | 103230154 103218702 103218549 | HSD11B1 MGST1 CBR3 | csab:103230154 csab:103218702 csab:103218549 |
| csab05202 | Transcriptional misregulation in cancer | 7/175 | 148/5900 | 0.149501079264612 | 0.49335356157322 | 103226390 103215685 103219133 103224652 103228427 103237430 103218917 | IL6 PLAT NR4A3 GADD45A NFKBIZ MYC TRAF1 | csab:103226390 csab:103215685 csab:103219133 csab:103224652 csab:103228427 csab:103237430 csab:103218917 |
| csab00601 | Glycosphingolipid biosynthesis - lacto and neolacto series | 2/175 | 24/5900 | 0.158146509469155 | 0.511343681625407 | 103234996 103217238 | FUT1 B3GALT1 | csab:103234996 csab:103217238 |
| csab05220 | Chronic myeloid leukemia | 4/175 | 71/5900 | 0.158826446565467 | 0.511343681625407 | 103224652 103246489 103237430 103224900 | GADD45A CCND1 MYC PIK3R3 | csab:103224652 csab:103246489 csab:103237430 csab:103224900 |
| csab01523 | Antifolate resistance | 2/175 | 25/5900 | 0.168720291327829 | 0.527919249517853 | 103226390 103241278 | IL6 IL1B | csab:103226390 csab:103241278 |
| csab04270 | Vascular smooth muscle contraction | 5/175 | 99/5900 | 0.169974000791733 | 0.527919249517853 | 103222156 103239748 103235528 103240389 103223064 | EDN1 CALCB PLA2G4C ADM ADORA2A | csab:103222156 csab:103239748 csab:103235528 csab:103240389 csab:103223064 |
| csab04931 | Insulin resistance | 5/175 | 99/5900 | 0.169974000791733 | 0.527919249517853 | 103226390 103247816 103224900 103214788 103220112 | IL6 TRIB3 PIK3R3 IRS2 GFPT1 | csab:103226390 csab:103247816 csab:103224900 csab:103214788 csab:103220112 |
| csab05226 | Gastric cancer | 6/175 | 127/5900 | 0.174815267025341 | 0.536642215054536 | 103224652 103246489 103223483 103237430 103224207 103224900 | GADD45A CCND1 WNT7B MYC WNT2B PIK3R3 | csab:103224652 csab:103246489 csab:103223483 csab:103237430 csab:103224207 csab:103224900 |
| csab04620 | Toll-like receptor signaling pathway | 4/175 | 75/5900 | 0.18211755302946 | 0.552632574710084 | 103226390 103241278 103230198 103224900 | IL6 IL1B IKBKE PIK3R3 | csab:103226390 csab:103241278 csab:103230198 csab:103224900 |
| csab04211 | Longevity regulating pathway | 4/175 | 76/5900 | 0.188111145441815 | 0.56419023195827 | 103240818 103225266 103224900 103214788 | SOD2 SESN2 PIK3R3 IRS2 | csab:103240818 csab:103225266 csab:103224900 csab:103214788 |
| csab04960 | Aldosterone-regulated sodium reabsorption | 2/175 | 27/5900 | 0.19020049486472 | 0.56419023195827 | 103224900 103229709 | PIK3R3 SCNN1B | csab:103224900 csab:103229709 |
| csab04350 | TGF-beta signaling pathway | 4/175 | 77/5900 | 0.194167838391478 | 0.569558992615003 | 103246725 103237430 103237062 103245923 | BMP2 MYC E2F5 GREM1 | csab:103246725 csab:103237430 csab:103237062 csab:103245923 |
| csab04150 | mTOR signaling pathway | 6/175 | 132/5900 | 0.197287881497175 | 0.572351656211585 | 103225266 103223483 103224207 103246611 103224900 103233435 | SESN2 WNT7B WNT2B CASTOR2 PIK3R3 SLC7A5 | csab:103225266 csab:103223483 csab:103224207 csab:103246611 csab:103224900 csab:103233435 |
| csab00250 | Alanine, aspartate and glutamate metabolism | 2/175 | 28/5900 | 0.201075617539145 | 0.576999598155808 | 103227954 103220112 | ABAT GFPT1 | csab:103227954 csab:103220112 |
| csab05133 | Pertussis | 3/175 | 53/5900 | 0.2072126734173 | 0.588216621313626 | 103226390 103241278 103221732 | IL6 IL1B LOC103221732 | csab:103226390 csab:103241278 csab:103221732 |
| csab00564 | Glycerophospholipid metabolism | 4/175 | 80/5900 | 0.212690172984788 | 0.597342613489191 | 103235528 103228224 103224742 103220573 | PLA2G4C PLA1A PLPP3 PLB1 | csab:103235528 csab:103228224 csab:103224742 csab:103220573 |
| csab05140 | Leishmaniasis | 3/175 | 54/5900 | 0.214976439205424 | 0.597408210002441 | 103241278 103218491 103223257 | IL1B PTPN6 NCF4 | csab:103241278 csab:103218491 csab:103223257 |
| csab05161 | Hepatitis B | 6/175 | 138/5900 | 0.225590414181652 | 0.620373638999543 | 103226390 103215465 103230198 103237430 103216128 103224900 | IL6 EGR3 IKBKE MYC EGR2 PIK3R3 | csab:103226390 csab:103215465 csab:103230198 csab:103237430 csab:103216128 csab:103224900 |
| csab05135 | Yersinia infection | 5/175 | 112/5900 | 0.238270510662136 | 0.642802109343594 | 103226390 103241278 103224900 103224044 103242710 | IL6 IL1B PIK3R3 PIP5K1A CCL2 | csab:103226390 csab:103241278 csab:103224900 csab:103224044 csab:103242710 |
| csab05221 | Acute myeloid leukemia | 3/175 | 57/5900 | 0.238615934529062 | 0.642802109343594 | 103246489 103237430 103224900 | CCND1 MYC PIK3R3 | csab:103246489 csab:103237430 csab:103224900 |
| csab05218 | Melanoma | 3/175 | 58/5900 | 0.246595232059451 | 0.657587285491869 | 103224652 103246489 103224900 | GADD45A CCND1 PIK3R3 | csab:103224652 csab:103246489 csab:103224900 |
| csab05142 | Chagas disease | 4/175 | 86/5900 | 0.251082866475525 | 0.662858767495386 | 103226390 103241278 103224900 103242710 | IL6 IL1B PIK3R3 CCL2 | csab:103226390 csab:103241278 csab:103224900 csab:103242710 |
| csab01522 | Endocrine resistance | 4/175 | 87/5900 | 0.25762587704219 | 0.673261771821698 | 103244654 103246489 103224900 103239621 | HBEGF CCND1 PIK3R3 NOTCH1 | csab:103244654 csab:103246489 csab:103224900 csab:103239621 |
| csab04721 | Synaptic vesicle cycle | 3/175 | 60/5900 | 0.262674100369829 | 0.673261771821698 | 103246629 103246502 103239806 | STX1A SNAP25 DNM1 | csab:103246629 csab:103246502 csab:103239806 |
| csab04917 | Prolactin signaling pathway | 3/175 | 60/5900 | 0.262674100369829 | 0.673261771821698 | 103246489 103238870 103224900 | CCND1 SOCS2 PIK3R3 | csab:103246489 csab:103238870 csab:103224900 |
| csab01200 | Carbon metabolism | 4/175 | 89/5900 | 0.270812352334354 | 0.687446740541051 | 103242625 103218486 103224094 103240824 | ALDOC ENO2 PHGDH ACAT2 | csab:103242625 csab:103218486 csab:103224094 csab:103240824 |
| csab05167 | Kaposi sarcoma-associated herpesvirus infection | 6/175 | 148/5900 | 0.275396750778699 | 0.692426116243585 | 103226390 103246489 103230198 103235789 103237430 103224900 | IL6 CCND1 IKBKE CXCL1 MYC PIK3R3 | csab:103226390 csab:103246489 csab:103230198 csab:103235798 csab:103237430 csab:103224900 |
| csab05214 | Glioma | 3/175 | 62/5900 | 0.278882484000754 | 0.694575243171689 | 103224652 103246489 103224900 | GADD45A CCND1 PIK3R3 | csab:103224652 csab:103246489 csab:103224900 |
| csab00430 | Taurine and hypotaurine metabolism | 1/175 | 11/5900 | 0.282148899886631 | 0.696143080094118 | 103230592 | FMO4 | csab:103230592 |
| csab00982 | Drug metabolism - cytochrome P450 | 2/175 | 36/5900 | 0.289557663221401 | 0.696774077323695 | 103218702 103230592 | MGST1 FMO4 | csab:103218702 csab:103230592 |
| csab04979 | Cholesterol metabolism | 2/175 | 36/5900 | 0.289557663221401 | 0.696774077323695 | 103234364 103229330 | LDLR NPC2 | csab:103234364 csab:103229330 |
| csab04810 | Regulation of actin cytoskeleton | 7/175 | 181/5900 | 0.290322532218206 | 0.696774077323695 | 103231401 103243277 103240591 103246479 103224900 103223856 103224044 | ITGAX ITGB3 WASF1 FGFR3 PIK3R3 IQGAP3 PIP5K1A | csab:103231401 csab:103243277 csab:103240591 csab:103246479 csab:103224900 csab:103223856 csab:103224044 |
| csab04911 | Insulin secretion | 3/175 | 64/5900 | 0.295184789703192 | 0.702061121456241 | 103226636 103246629 103246502 | PCLO STX1A SNAP25 | csab:103226636 csab:103246629 csab:103246502 |
| csab00260 | Glycine, serine and threonine metabolism | 2/175 | 37/5900 | 0.300634238083933 | 0.703283768912283 | 103224624 103224094 | CTH PHGDH | csab:103224624 csab:103224094 |
| csab04014 | Ras signaling pathway | 7/175 | 184/5900 | 0.304558577216257 | 0.703283768912283 | 103230197 103225530 103246479 103235528 103228224 103224900 103223351 | RASSF5 EPHA2 FGFR3 PLA2G4C PLA1A PIK3R3 RIN1 | csab:103230197 csab:103225530 csab:103246479 csab:103235528 csab:103228224 csab:103224900 csab:103223351 |
| csab05100 | Bacterial invasion of epithelial cells | 3/175 | 66/5900 | 0.311547106500921 | 0.703283768912283 | 103240591 103224900 103239806 | WASF1 PIK3R3 DNM1 | csab:103240591 csab:103224900 csab:103239806 |
| csab00380 | Tryptophan metabolism | 2/175 | 38/5900 | 0.311682579404307 | 0.703283768912283 | 103240824 103232876 | ACAT2 LOC103232876 | csab:103240824 csab:103232876 |
| csab04961 | Endocrine and other factor-regulated calcium reabsorption | 2/175 | 38/5900 | 0.311682579404307 | 0.703283768912283 | 103238835 103239806 | ATP2B1 DNM1 | csab:103238835 csab:103239806 |
| csab05204 | Chemical carcinogenesis - DNA adducts | 2/175 | 38/5900 | 0.311682579404307 | 0.703283768912283 | 103230154 103218702 | HSD11B1 MGST1 | csab:103230154 csab:103218702 |
| csab04727 | GABAergic synapse | 3/175 | 67/5900 | 0.319740642402436 | 0.713821152742699 | 103238195 103227954 103238196 | SLC38A1 ABAT SLC38A2 | csab:103238195 csab:103227954 csab:103238196 |
| csab00140 | Steroid hormone biosynthesis | 2/175 | 39/5900 | 0.322694652229379 | 0.713821152742699 | 103230154 103237812 | HSD11B1 LOC103237812 | csab:103230154 csab:103237812 |
| csab04934 | Cushing syndrome | 5/175 | 127/5900 | 0.324464160337591 | 0.713821152742699 | 103238335 103246489 103223483 103234364 103224207 | NR4A1 CCND1 WNT7B LDLR WNT2B | csab:103238335 csab:103246489 csab:103223483 csab:103234364 csab:103224207 |
| csab04072 | Phospholipase D signaling pathway | 5/175 | 128/5900 | 0.330368311489987 | 0.720803588705427 | 103235528 103224742 103224900 103224044 103239806 | PLA2G4C PLPP3 PIK3R3 PIP5K1A DNM1 | csab:103235528 csab:103224742 csab:103224900 csab:103224044 csab:103239806 |
| csab03018 | RNA degradation | 3/175 | 69/5900 | 0.336133170172623 | 0.727213917862033 | 103218486 103242916 103234733 | ENO2 TOB1 EXOSC5 | csab:103218486 csab:103242916 csab:103234733 |
| csab01210 | 2-Oxocarboxylic acid metabolism | 1/175 | 14/5900 | 0.344272137424949 | 0.727213917862033 | 103218755 | BCAT1 | csab:103218755 |
| csab04977 | Vitamin digestion and absorption | 1/175 | 14/5900 | 0.344272137424949 | 0.727213917862033 | 103220573 | PLB1 | csab:103220573 |
| csab04925 | Aldosterone synthesis and secretion | 3/175 | 70/5900 | 0.344324771714978 | 0.727213917862033 | 103238335 103234364 103238835 | NR4A1 LDLR ATP2B1 | csab:103238335 csab:103234364 csab:103238835 |
| csab04380 | Osteoclast differentiation | 4/175 | 101/5900 | 0.351715265562324 | 0.73028922005502 | 103241278 103243277 103223257 103224900 | IL1B ITGB3 NCF4 PIK3R3 | csab:103241278 csab:103243277 csab:103223257 csab:103224900 |
| csab04670 | Leukocyte transendothelial migration | 4/175 | 101/5900 | 0.351715265562324 | 0.73028922005502 | 103230197 103241901 103223257 103224900 | RASSF5 CLDN1 NCF4 PIK3R3 | csab:103230197 csab:103241901 csab:103223257 csab:103224900 |
| csab04932 | Non-alcoholic fatty liver disease | 5/175 | 132/5900 | 0.354079621844858 | 0.73028922005502 | 103226390 103241278 103224900 103214788 103223920 | IL6 IL1B PIK3R3 IRS2 IL6R | csab:103226390 csab:103241278 csab:103224900 csab:103214788 csab:103223920 |
| csab04612 | Antigen processing and presentation | 2/175 | 43/5900 | 0.366236463742691 | 0.743308912133936 | 103223677 103221664 | HSPA6 LOC103221664 | csab:103223677 csab:103221664 |
| csab05321 | Inflammatory bowel disease | 2/175 | 43/5900 | 0.366236463742691 | 0.743308912133936 | 103226390 103241278 | IL6 IL1B | csab:103226390 csab:103241278 |
| csab05212 | Pancreatic cancer | 3/175 | 73/5900 | 0.368838892005855 | 0.743308912133936 | 103224652 103246489 103224900 | GADD45A CCND1 PIK3R3 | csab:103224652 csab:103246489 csab:103224900 |
| csab00480 | Glutathione metabolism | 2/175 | 44/5900 | 0.376963276914195 | 0.753926553828389 | 103220822 103218702 | ODC1 MGST1 | csab:103220822 csab:103218702 |
| csab00603 | Glycosphingolipid biosynthesis - globo and isoglobo series | 1/175 | 16/5900 | 0.382687343575556 | 0.757686909831778 | 103234996 | FUT1 | csab:103234996 |
| csab04974 | Protein digestion and absorption | 3/175 | 75/5900 | 0.385098226296727 | 0.757686909831778 | 103214795 103242775 103238196 | COL4A1 COL20A1 SLC38A2 | csab:103214795 csab:103242775 csab:103238196 |
| csab04152 | AMPK signaling pathway | 4/175 | 107/5900 | 0.392493108279803 | 0.757686909831778 | 103246489 103224900 103223243 103214788 | CCND1 PIK3R3 HMGCR IRS2 | csab:103246489 csab:103224900 csab:103223243 csab:103214788 |
| csab04916 | Melanogenesis | 3/175 | 76/5900 | 0.393193585783915 | 0.757686909831778 | 103222156 103223483 103224207 | EDN1 WNT7B WNT2B | csab:103222156 csab:103223483 csab:103224207 |
| csab05414 | Dilated cardiomyopathy | 3/175 | 76/5900 | 0.393193585783915 | 0.757686909831778 | 103243277 103217090 103219220 | ITGB3 CACNB4 TPM2 | csab:103243277 csab:103217090 csab:103219220 |
| csab00532 | Glycosaminoglycan biosynthesis - chondroitin sulfate / dermatan sulfate | 1/175 | 17/5900 | 0.401047254329172 | 0.764106963389091 | 103215414 | CSGALNACT1 | csab:103215414 |
| csab04015 | Rap1 signaling pathway | 6/175 | 172/5900 | 0.402313893602589 | 0.764106963389091 | 103243277 103230197 103225530 103246479 103223064 103224900 | ITGB3 RASSF5 EPHA2 FGFR3 ADORA2A PIK3R3 | csab:103243277 csab:103230197 csab:103225530 csab:103246479 csab:103223064 csab:103224900 |
| csab05150 | Staphylococcus aureus infection | 2/175 | 47/5900 | 0.408675262026855 | 0.766351342253731 | 103221732 103233571 | LOC103221732 CFD | csab:103221732 csab:103233571 |
| csab04750 | Inflammatory mediator regulation of TRP channels | 3/175 | 78/5900 | 0.409301285067334 | 0.766351342253731 | 103241278 103235528 103224900 | IL1B PLA2G4C PIK3R3 | csab:103241278 csab:103235528 csab:103224900 |
| csab04923 | Regulation of lipolysis in adipocytes | 2/175 | 48/5900 | 0.419074294148489 | 0.769080070635229 | 103224900 103214788 | PIK3R3 IRS2 | csab:103224900 csab:103214788 |
| csab04110 | Cell cycle | 4/175 | 111/5900 | 0.419498220346489 | 0.769080070635229 | 103224652 103246489 103237430 103237062 | GADD45A CCND1 MYC E2F5 | csab:103224652 csab:103246489 csab:103237430 csab:103237062 |
| csab04910 | Insulin signaling pathway | 4/175 | 111/5900 | 0.419498220346489 | 0.769080070635229 | 103238870 103224900 103214788 103228406 | SOCS2 PIK3R3 IRS2 CBLB | csab:103238870 csab:103224900 csab:103214788 csab:103228406 |
| csab04660 | T cell receptor signaling pathway | 3/175 | 80/5900 | 0.425280308321451 | 0.772762263685426 | 103218491 103224900 103228406 | PTPN6 PIK3R3 CBLB | csab:103218491 csab:103224900 csab:103228406 |
| csab04370 | VEGF signaling pathway | 2/175 | 49/5900 | 0.429380619551337 | 0.772762263685426 | 103235528 103224900 | PLA2G4C PIK3R3 | csab:103235528 csab:103224900 |
| csab04512 | ECM-receptor interaction | 3/175 | 81/5900 | 0.433215208429709 | 0.772762263685426 | 103243277 103220724 103214795 | ITGB3 SDC1 COL4A1 | csab:103243277 csab:103220724 csab:103214795 |
| csab04659 | Th17 cell differentiation | 3/175 | 81/5900 | 0.433215208429709 | 0.772762263685426 | 103226390 103241278 103223920 | IL6 IL1B IL6R | csab:103226390 csab:103241278 csab:103223920 |
| csab05320 | Autoimmune thyroid disease | 1/175 | 19/5900 | 0.436153967920355 | 0.772782869335395 | 103237469 | TG | csab:103237469 |
| csab04927 | Cortisol synthesis and secretion | 2/175 | 50/5900 | 0.439590578198829 | 0.77367941762994 | 103238335 103234364 | NR4A1 LDLR | csab:103238335 csab:103234364 |
| csab04144 | Endocytosis | 7/175 | 214/5900 | 0.451093038930038 | 0.784654159482918 | 103246479 103234364 103224044 103233004 103223677 103228406 103239806 | FGFR3 LDLR PIP5K1A EHD1 HSPA6 CBLB DNM1 | csab:103246479 csab:103234364 csab:103224044 csab:103233004 csab:103223677 csab:103228406 csab:103239806 |
| csab05165 | Human papillomavirus infection | 9/175 | 281/5900 | 0.455801186343053 | 0.784654159482918 | 103243277 103248148 103246489 103223483 103230198 103224207 103214795 103224900 103239621 | ITGB3 DLG2 CCND1 WNT7B IKBKE WNT2B COL4A1 PIK3R3 NOTCH1 | csab:103243277 csab:103248148 csab:103246489 csab:103223483 csab:103230198 csab:103224207 csab:103214795 csab:103224900 csab:103239621 |
| csab04340 | Hedgehog signaling pathway | 2/175 | 52/5900 | 0.459708220011371 | 0.784654159482918 | 103219690 103246489 | GAS1 CCND1 | csab:103219690 csab:103246489 |
| csab04976 | Bile secretion | 2/175 | 52/5900 | 0.459708220011371 | 0.784654159482918 | 103234364 103223243 | LDLR HMGCR | csab:103234364 csab:103223243 |
| csab05166 | Human T-cell leukemia virus 1 infection | 6/175 | 183/5900 | 0.460897836751128 | 0.784654159482918 | 103226390 103246489 103236305 103237430 103216128 103224900 | IL6 CCND1 IL15 MYC EGR2 PIK3R3 | csab:103226390 csab:103246489 csab:103236305 csab:103237430 csab:103216128 csab:103224900 |
| csab05215 | Prostate cancer | 3/175 | 85/5900 | 0.464536643183971 | 0.784654159482918 | 103215685 103246489 103224900 | PLAT CCND1 PIK3R3 | csab:103215685 csab:103246489 csab:103224900 |
| csab00010 | Glycolysis / Gluconeogenesis | 2/175 | 53/5900 | 0.469610006152314 | 0.784654159482918 | 103242625 103218486 | ALDOC ENO2 | csab:103242625 csab:103218486 |
| csab04970 | Salivary secretion | 2/175 | 53/5900 | 0.469610006152314 | 0.784654159482918 | 103238835 103244467 | ATP2B1 SLC12A2 | csab:103238835 csab:103244467 |
| csab04142 | Lysosome | 4/175 | 119/5900 | 0.472575800597666 | 0.784654159482918 | 103242313 103245241 103229330 103243148 | CD68 CTSH NPC2 ARSG | csab:103242313 csab:103245241 csab:103229330 csab:103243148 |
| csab04664 | Fc epsilon RI signaling pathway | 2/175 | 54/5900 | 0.479403604881041 | 0.785544007076793 | 103235528 103224900 | PLA2G4C PIK3R3 | csab:103235528 csab:103224900 |
| csab04724 | Glutamatergic synapse | 3/175 | 87/5900 | 0.479917052892836 | 0.785544007076793 | 103235528 103238195 103238196 | PLA2G4C SLC38A1 SLC38A2 | csab:103235528 csab:103238195 csab:103238196 |
| csab00515 | Mannose type O-glycan biosynthesis | 1/175 | 22/5900 | 0.485013913460293 | 0.785544007076793 | 103242981 | MGAT5B | csab:103242981 |
| csab00790 | Folate biosynthesis | 1/175 | 22/5900 | 0.485013913460293 | 0.785544007076793 | 103237812 | LOC103237812 | csab:103237812 |
| csab05203 | Viral carcinogenesis | 5/175 | 155/5900 | 0.489415058468355 | 0.787838874607597 | 103215465 103246489 103218917 103216128 103224900 | EGR3 CCND1 TRAF1 EGR2 PIK3R3 | csab:103215465 csab:103246489 csab:103218917 csab:103216128 csab:103224900 |
| csab00630 | Glyoxylate and dicarboxylate metabolism | 1/175 | 23/5900 | 0.500346095349447 | 0.800553752559115 | 103240824 | ACAT2 | csab:103240824 |
| csab04662 | B cell receptor signaling pathway | 2/175 | 57/5900 | 0.508113249384928 | 0.803244897231264 | 103218491 103224900 | PTPN6 PIK3R3 | csab:103218491 csab:103224900 |
| csab04920 | Adipocytokine signaling pathway | 2/175 | 57/5900 | 0.508113249384928 | 0.803244897231264 | 103225587 103214788 | TNFRSF1B IRS2 | csab:103225587 csab:103214788 |
| csab00030 | Pentose phosphate pathway | 1/175 | 24/5900 | 0.515224338213807 | 0.809638245764554 | 103242625 | ALDOC | csab:103242625 |
| csab01524 | Platinum drug resistance | 2/175 | 61/5900 | 0.544762574477392 | 0.841036957087903 | 103218702 103224900 | MGST1 PIK3R3 | csab:103218702 csab:103224900 |
| csab04260 | Cardiac muscle contraction | 2/175 | 61/5900 | 0.544762574477392 | 0.841036957087903 | 103217090 103219220 | CACNB4 TPM2 | csab:103217090 csab:103219220 |
| csab05211 | Renal cell carcinoma | 2/175 | 61/5900 | 0.544762574477392 | 0.841036957087903 | 103220342 103224900 | EPAS1 PIK3R3 | csab:103220342 csab:103224900 |
| csab04062 | Chemokine signaling pathway | 4/175 | 133/5900 | 0.560615036990077 | 0.855689120881966 | 103235789 103242198 103224900 103242710 | CXCL1 CXCL16 PIK3R3 CCL2 | csab:103235798 csab:103242198 csab:103224900 csab:103242710 |
| csab04972 | Pancreatic secretion | 2/175 | 63/5900 | 0.56236626672158 | 0.855689120881966 | 103238835 103244467 | ATP2B1 SLC12A2 | csab:103238835 csab:103244467 |
| csab00410 | beta-Alanine metabolism | 1/175 | 28/5900 | 0.570459413921311 | 0.855689120881966 | 103227954 | ABAT | csab:103227954 |
| csab00640 | Propanoate metabolism | 1/175 | 28/5900 | 0.570459413921311 | 0.855689120881966 | 103227954 | ABAT | csab:103227954 |
| csab04710 | Circadian rhythm | 1/175 | 28/5900 | 0.570459413921311 | 0.855689120881966 | 103227939 | BHLHE40 | csab:103227939 |
| csab04935 | Growth hormone synthesis, secretion and action | 3/175 | 102/5900 | 0.587872558094443 | 0.867256402697243 | 103238870 103224900 103214788 | SOCS2 PIK3R3 IRS2 | csab:103238870 csab:103224900 csab:103214788 |
| csab04915 | Estrogen signaling pathway | 3/175 | 103/5900 | 0.594549029082107 | 0.867256402697243 | 103244654 103224900 103223677 | HBEGF PIK3R3 HSPA6 | csab:103244654 csab:103224900 csab:103223677 |
| csab04520 | Adherens junction | 2/175 | 67/5900 | 0.596103617189291 | 0.867256402697243 | 103240591 103218491 | WASF1 PTPN6 | csab:103240591 csab:103218491 |
| csab04650 | Natural killer cell mediated cytotoxicity | 2/175 | 67/5900 | 0.596103617189291 | 0.867256402697243 | 103218491 103224900 | PTPN6 PIK3R3 | csab:103218491 csab:103224900 |
| csab05020 | Prion disease | 6/175 | 211/5900 | 0.601287280068963 | 0.867256402697243 | 103226390 103241278 103223257 103224900 103239621 103223677 | IL6 IL1B NCF4 PIK3R3 NOTCH1 HSPA6 | csab:103226390 csab:103241278 csab:103223257 csab:103224900 csab:103239621 csab:103223677 |
| csab05412 | Arrhythmogenic right ventricular cardiomyopathy | 2/175 | 68/5900 | 0.604229870831284 | 0.867256402697243 | 103243277 103217090 | ITGB3 CACNB4 | csab:103243277 csab:103217090 |
| csab04217 | Necroptosis | 3/175 | 105/5900 | 0.607691992941025 | 0.867256402697243 | 103241278 103235528 103240296 | IL1B PLA2G4C TNFAIP3 | csab:103241278 csab:103235528 csab:103240296 |
| csab04611 | Platelet activation | 3/175 | 105/5900 | 0.607691992941025 | 0.867256402697243 | 103243277 103235528 103224900 | ITGB3 PLA2G4C PIK3R3 | csab:103243277 csab:103235528 csab:103224900 |
| csab04973 | Carbohydrate digestion and absorption | 1/175 | 31/5900 | 0.607736494314356 | 0.867256402697243 | 103224900 | PIK3R3 | csab:103224900 |
| csab04514 | Cell adhesion molecules | 3/175 | 106/5900 | 0.614157444523743 | 0.870796141866486 | 103220287 103220724 103241901 | NRXN1 SDC1 CLDN1 | csab:103220287 csab:103220724 csab:103241901 |
| csab00051 | Fructose and mannose metabolism | 1/175 | 32/5900 | 0.61943288441403 | 0.870796141866486 | 103242625 | ALDOC | csab:103242625 |
| csab01232 | Nucleotide metabolism | 2/175 | 70/5900 | 0.620112404056437 | 0.870796141866486 | 103225017 103240330 | CTPS1 AMPD3 | csab:103225017 csab:103240330 |
| csab04130 | SNARE interactions in vesicular transport | 1/175 | 33/5900 | 0.630782449040401 | 0.87992322306581 | 103246629 | STX1A | csab:103246629 |
| csab04510 | Focal adhesion | 5/175 | 182/5900 | 0.633278077206454 | 0.87992322306581 | 103243277 103246489 103214795 103224900 103224044 | ITGB3 CCND1 COL4A1 PIK3R3 PIP5K1A | csab:103243277 csab:103246489 csab:103214795 csab:103224900 csab:103224044 |
| csab04912 | GnRH signaling pathway | 2/175 | 73/5900 | 0.643014646848515 | 0.883359852623121 | 103244654 103235528 | HBEGF PLA2G4C | csab:103244654 csab:103235528 |
| csab04926 | Relaxin signaling pathway | 3/175 | 111/5900 | 0.64540847257791 | 0.883359852623121 | 103222156 103214795 103224900 | EDN1 COL4A1 PIK3R3 | csab:103222156 csab:103214795 csab:103224900 |
| csab05235 | PD-L1 expression and PD-1 checkpoint pathway in cancer | 2/175 | 74/5900 | 0.65040452594525 | 0.883359852623121 | 103218491 103224900 | PTPN6 PIK3R3 | csab:103218491 csab:103224900 |
| csab00620 | Pyruvate metabolism | 1/175 | 35/5900 | 0.652481709323896 | 0.883359852623121 | 103240824 | ACAT2 | csab:103240824 |
| csab01250 | Biosynthesis of nucleotide sugars | 1/175 | 35/5900 | 0.652481709323896 | 0.883359852623121 | 103220112 | GFPT1 | csab:103220112 |
| csab04978 | Mineral absorption | 1/175 | 36/5900 | 0.662850967783968 | 0.892209584763505 | 103238835 | ATP2B1 | csab:103238835 |
| csab04261 | Adrenergic signaling in cardiomyocytes | 3/175 | 115/5900 | 0.669102927820864 | 0.892209584763505 | 103217090 103219220 103238835 | CACNB4 TPM2 ATP2B1 | csab:103217090 csab:103219220 csab:103238835 |
| csab00071 | Fatty acid degradation | 1/175 | 37/5900 | 0.672912543608969 | 0.892209584763505 | 103240824 | ACAT2 | csab:103240824 |
| csab03050 | Proteasome | 1/175 | 37/5900 | 0.672912543608969 | 0.892209584763505 | 103233253 | PSMB10 | csab:103233253 |
| csab05168 | Herpes simplex virus 1 infection | 11/175 | 410/5900 | 0.68071138171445 | 0.892209584763505 | 103226390 103241278 103243277 103239154 103230198 103224900 103232396 103221664 103219115 103242710 103239156 | IL6 IL1B ITGB3 OAS1 IKBKE PIK3R3 NXF3 LOC103221664 ZNF189 CCL2 OAS2 | csab:103226390 csab:103241278 csab:103243277 csab:103239154 csab:103230198 csab:103224900 csab:103232396 csab:103221664 csab:103219115 csab:103242710 csab:103239156 |
| csab00330 | Arginine and proline metabolism | 1/175 | 38/5900 | 0.6826755156145 | 0.892209584763505 | 103220822 | ODC1 | csab:103220822 |
| csab00514 | Other types of O-glycan biosynthesis | 1/175 | 38/5900 | 0.6826755156145 | 0.892209584763505 | 103227904 | GXYLT2 | csab:103227904 |
| csab04613 | Neutrophil extracellular trap formation | 3/175 | 118/5900 | 0.686107448846576 | 0.892277667465497 | 103243277 103223257 103224900 | ITGB3 NCF4 PIK3R3 | csab:103243277 csab:103223257 csab:103224900 |
| csab05416 | Viral myocarditis | 1/175 | 40/5900 | 0.701340639270295 | 0.903120471627095 | 103246489 | CCND1 | csab:103246489 |
| csab01240 | Biosynthesis of cofactors | 3/175 | 121/5900 | 0.702456579755293 | 0.903120471627095 | 103218755 103225017 103225584 | BCAT1 CTPS1 DHRS3 | csab:103218755 csab:103225017 csab:103225584 |
| csab04921 | Oxytocin signaling pathway | 3/175 | 122/5900 | 0.707761327831767 | 0.903120471627095 | 103246489 103217090 103235528 | CCND1 CACNB4 PLA2G4C | csab:103246489 csab:103217090 csab:103235528 |
| csab04024 | cAMP signaling pathway | 4/175 | 161/5900 | 0.709852439487527 | 0.903120471627095 | 103222156 103223064 103224900 103238835 | EDN1 ADORA2A PIK3R3 ATP2B1 | csab:103222156 csab:103223064 csab:103224900 csab:103238835 |
| csab04070 | Phosphatidylinositol signaling system | 2/175 | 83/5900 | 0.711549462494075 | 0.903120471627095 | 103224900 103224044 | PIK3R3 PIP5K1A | csab:103224900 csab:103224044 |
| csab00520 | Amino sugar and nucleotide sugar metabolism | 1/175 | 44/5900 | 0.735458477015876 | 0.924285789661704 | 103220112 | GFPT1 | csab:103220112 |
| csab04742 | Taste transduction | 1/175 | 44/5900 | 0.735458477015876 | 0.924285789661704 | 103229709 | SCNN1B | csab:103229709 |
| csab05152 | Tuberculosis | 3/175 | 129/5900 | 0.742890988427485 | 0.924285789661704 | 103226390 103241278 103231401 | IL6 IL1B ITGAX | csab:103226390 csab:103241278 csab:103231401 |
| csab00510 | N-Glycan biosynthesis | 1/175 | 46/5900 | 0.751034593203769 | 0.924285789661704 | 103242981 | MGAT5B | csab:103242981 |
| csab00983 | Drug metabolism - other enzymes | 1/175 | 46/5900 | 0.751034593203769 | 0.924285789661704 | 103218702 | MGST1 | csab:103218702 |
| csab04730 | Long-term depression | 1/175 | 46/5900 | 0.751034593203769 | 0.924285789661704 | 103235528 | PLA2G4C | csab:103235528 |
| csab05145 | Toxoplasmosis | 2/175 | 90/5900 | 0.752732745368433 | 0.924285789661704 | 103234364 103223677 | LDLR HSPA6 | csab:103234364 csab:103223677 |
| csab00561 | Glycerolipid metabolism | 1/175 | 47/5900 | 0.758477187359789 | 0.927027673439742 | 103224742 | PLPP3 | csab:103224742 |
| csab05031 | Amphetamine addiction | 1/175 | 48/5900 | 0.765698525513221 | 0.927491413110432 | 103246629 | STX1A | csab:103246629 |
| csab04530 | Tight junction | 3/175 | 134/5900 | 0.765883060826039 | 0.927491413110432 | 103248148 103246489 103241901 | DLG2 CCND1 CLDN1 | csab:103248148 csab:103246489 csab:103241901 |
| csab00240 | Pyrimidine metabolism | 1/175 | 49/5900 | 0.772705148554093 | 0.927722245743829 | 103225017 | CTPS1 | csab:103225017 |
| csab04020 | Calcium signaling pathway | 4/175 | 176/5900 | 0.773101871453191 | 0.927722245743829 | 103215153 103246479 103223064 103238835 | GDNF FGFR3 ADORA2A ATP2B1 | csab:103215153 csab:103246479 csab:103223064 csab:103238835 |
| csab01212 | Fatty acid metabolism | 1/175 | 50/5900 | 0.779503405091956 | 0.930628953668904 | 103240824 | ACAT2 | csab:103240824 |
| csab04330 | Notch signaling pathway | 1/175 | 51/5900 | 0.786099457076385 | 0.930628953668904 | 103239621 | NOTCH1 | csab:103239621 |
| csab04924 | Renin secretion | 1/175 | 51/5900 | 0.786099457076385 | 0.930628953668904 | 103222156 | EDN1 | csab:103222156 |
| csab04622 | RIG-I-like receptor signaling pathway | 1/175 | 52/5900 | 0.79249928525413 | 0.93300484663475 | 103230198 | IKBKE | csab:103230198 |
| csab03320 | PPAR signaling pathway | 1/175 | 53/5900 | 0.798708694467626 | 0.93300484663475 | 103215216 | HMGCS1 | csab:103215216 |
| csab04929 | GnRH secretion | 1/175 | 53/5900 | 0.798708694467626 | 0.93300484663475 | 103224900 | PIK3R3 | csab:103224900 |
| csab04145 | Phagosome | 2/175 | 100/5900 | 0.802765845383919 | 0.933613141768082 | 103243277 103223257 | ITGB3 NCF4 | csab:103243277 csab:103223257 |
| csab04071 | Sphingolipid signaling pathway | 2/175 | 102/5900 | 0.811630816975809 | 0.936912957589813 | 103224900 103230017 | PIK3R3 DEGS1 | csab:103224900 csab:103230017 |
| csab05208 | Chemical carcinogenesis - reactive oxygen species | 4/175 | 188/5900 | 0.815535736886172 | 0.936912957589813 | 103240818 103218702 103224900 103237812 | SOD2 MGST1 PIK3R3 LOC103237812 | csab:103240818 csab:103218702 csab:103224900 csab:103237812 |
| csab04918 | Thyroid hormone synthesis | 1/175 | 56/5900 | 0.816249925172943 | 0.936912957589813 | 103237469 | TG | csab:103237469 |
| csab00310 | Lysine degradation | 1/175 | 57/5900 | 0.821752365812015 | 0.939145560928017 | 103240824 | ACAT2 | csab:103240824 |
| csab05132 | Salmonella infection | 4/175 | 199/5900 | 0.848546432723913 | 0.954878069168623 | 103226390 103241278 103237430 103220717 | IL6 IL1B MYC RHOB | csab:103226390 csab:103241278 csab:103237430 csab:103220717 |
| csab05170 | Human immunodeficiency virus 1 infection | 3/175 | 157/5900 | 0.851027000165132 | 0.954878069168623 | 103225587 103224900 103221664 | TNFRSF1B PIK3R3 LOC103221664 | csab:103225587 csab:103224900 csab:103221664 |
| csab05322 | Systemic lupus erythematosus | 1/175 | 63/5900 | 0.851490901752415 | 0.954878069168623 | 103221732 | LOC103221732 | csab:103221732 |
| csab04371 | Apelin signaling pathway | 2/175 | 113/5900 | 0.854332811388242 | 0.954878069168623 | 103215685 103246489 | PLAT CCND1 | csab:103215685 csab:103246489 |
| csab04360 | Axon guidance | 3/175 | 161/5900 | 0.862732193126363 | 0.954878069168623 | 103225530 103246295 103224900 | EPHA2 SLIT2 PIK3R3 | csab:103225530 csab:103246295 csab:103224900 |
| csab03008 | Ribosome biogenesis in eukaryotes | 1/175 | 66/5900 | 0.864454009588261 | 0.954878069168623 | 103232396 | NXF3 | csab:103232396 |
| csab04146 | Peroxisome | 1/175 | 66/5900 | 0.864454009588261 | 0.954878069168623 | 103240818 | SOD2 | csab:103240818 |
| csab04740 | Olfactory transduction | 1/175 | 66/5900 | 0.864454009588261 | 0.954878069168623 | 103218440 | ANO2 | csab:103218440 |
| csab04658 | Th1 and Th2 cell differentiation | 1/175 | 69/5900 | 0.876291490453093 | 0.960004615457076 | 103239621 | NOTCH1 | csab:103239621 |
| csab04080 | Neuroactive ligand-receptor interaction | 4/175 | 210/5900 | 0.876461193443913 | 0.960004615457076 | 103222156 103239748 103240389 103223064 | EDN1 CALCB ADM ADORA2A | csab:103222156 csab:103239748 csab:103240389 csab:103223064 |
| csab00562 | Inositol phosphate metabolism | 1/175 | 70/5900 | 0.880004230835653 | 0.960004615457076 | 103224044 | PIP5K1A | csab:103224044 |
| csab05415 | Diabetic cardiomyopathy | 3/175 | 169/5900 | 0.883755679923675 | 0.9601296275714 | 103223257 103224900 103220112 | NCF4 PIK3R3 GFPT1 | csab:103223257 csab:103224900 csab:103220112 |
| csab05010 | Alzheimer disease | 6/175 | 302/5900 | 0.891640124482355 | 0.961486609930059 | 103226390 103241278 103223483 103224207 103224900 103214788 | IL6 IL1B WNT7B WNT2B PIK3R3 IRS2 | csab:103226390 csab:103241278 csab:103223483 csab:103224207 csab:103224900 csab:103214788 |
| csab04140 | Autophagy - animal | 2/175 | 126/5900 | 0.893368151662082 | 0.961486609930059 | 103224900 103214788 | PIK3R3 IRS2 | csab:103224900 csab:103214788 |
| csab04022 | cGMP-PKG signaling pathway | 2/175 | 127/5900 | 0.895930704707555 | 0.961486609930059 | 103238835 103214788 | ATP2B1 IRS2 | csab:103238835 csab:103214788 |
| csab04120 | Ubiquitin mediated proteolysis | 2/175 | 130/5900 | 0.903280387882535 | 0.965449483404815 | 103227647 103228406 | UBA7 CBLB | csab:103227647 csab:103228406 |
| csab03015 | mRNA surveillance pathway | 1/175 | 79/5900 | 0.908807451508886 | 0.966461616265343 | 103232396 | NXF3 | csab:103232396 |
| csab04914 | Progesterone-mediated oocyte maturation | 1/175 | 80/5900 | 0.911549024432085 | 0.966461616265343 | 103224900 | PIK3R3 | csab:103224900 |
| csab04726 | Serotonergic synapse | 1/175 | 84/5900 | 0.921719680745777 | 0.973335982867541 | 103235528 | PLA2G4C | csab:103235528 |
| csab03013 | Nucleocytoplasmic transport | 1/175 | 89/5900 | 0.932812586894976 | 0.973451766503826 | 103232396 | NXF3 | csab:103232396 |
| csab04725 | Cholinergic synapse | 1/175 | 89/5900 | 0.932812586894976 | 0.973451766503826 | 103224900 | PIK3R3 | csab:103224900 |
| csab05022 | Pathways of neurodegeneration - multiple diseases | 7/175 | 373/5900 | 0.933275459460291 | 0.973451766503826 | 103226390 103241278 103223483 103225587 103224207 103246629 103227647 | IL6 IL1B WNT7B TNFRSF1B WNT2B STX1A UBA7 | csab:103226390 csab:103241278 csab:103223483 csab:103225587 csab:103224207 csab:103246629 csab:103227647 |
| csab04141 | Protein processing in endoplasmic reticulum | 2/175 | 147/5900 | 0.936578593530196 | 0.973451766503826 | 103233048 103223677 | HERPUD1 HSPA6 | csab:103233048 csab:103223677 |
| csab04928 | Parathyroid hormone synthesis, secretion and action | 1/175 | 93/5900 | 0.940549648514567 | 0.973581626295225 | 103244654 | HBEGF | csab:103244654 |
| csab03010 | Ribosome | 1/175 | 95/5900 | 0.944079152771128 | 0.973581626295225 | 103221259 | RPL22L1 | csab:103221259 |
| csab04722 | Neurotrophin signaling pathway | 1/175 | 102/5900 | 0.954869716054867 | 0.97861220506367 | 103224900 | PIK3R3 | csab:103224900 |
| csab03040 | Spliceosome | 1/175 | 104/5900 | 0.957553148751417 | 0.97861220506367 | 103223677 | HSPA6 | csab:103223677 |
| csab00230 | Purine metabolism | 1/175 | 106/5900 | 0.96007788299807 | 0.97861220506367 | 103240330 | AMPD3 | csab:103240330 |
| csab05017 | Spinocerebellar ataxia | 1/175 | 114/5900 | 0.968768526770029 | 0.981785992070256 | 103224900 | PIK3R3 | csab:103224900 |
| csab05034 | Alcoholism | 1/175 | 116/5900 | 0.970629333069458 | 0.981785992070256 | 103223064 | ADORA2A | csab:103223064 |
| csab05016 | Huntington disease | 3/175 | 242/5900 | 0.977585102836734 | 0.985047584537778 | 103240818 103246306 103246629 | SOD2 TGM2 STX1A | csab:103240818 csab:103246306 csab:103246629 |
| csab05012 | Parkinson disease | 2/175 | 198/5900 | 0.983120255595302 | 0.986858355426463 | 103223064 103227647 | ADORA2A UBA7 | csab:103223064 csab:103227647 |
| csab05014 | Amyotrophic lateral sclerosis | 2/175 | 284/5900 | 0.998420505014975 | 0.998420505014975 | 103225587 103232396 | TNFRSF1B NXF3 | csab:103225587 csab:103232396 |
